# Frontiers in Toxicogenomics in the Twenty-First Century—the Grand Challenge: To Understand How the Genome and Epigenome Interact with the Toxic Environment at the Single-Cell, Whole-Organism, and Multi-Generational Level

**DOI:** 10.3389/fgene.2017.00173

**Published:** 2017-11-09

**Authors:** Douglas M. Ruden, Katherine Gurdziel, Michael Aschner

**Affiliations:** ^1^Department of Obstetrics and Gynecology, Wayne State University, Detroit, MI, United States; ^2^C. S. Mott Center for Human Health and Development, Wayne State University, Detroit, MI, United States; ^3^Institute of Environmental Health Sciences, Wayne State University, Detroit, MI, United States; ^4^Department of Molecular Pharmacology, Albert Einstein College of Medicine, Bronx, NY, United States

**Keywords:** toxicogenomics, RNA-Seq, ScRNA-seq, organoids, multigenerational epigenetics, transgenerational epigenetics, ATAC-seq

## Introduction

In 2011, we wrote the inaugural Grand Challenge for Frontiers in Toxicogenomics, a specialty section of Frontiers in Genetics. In the original Grand Challenge, we argued that that the fields of “Genomics” and “Toxicology” needed to be merged into the new field of “Toxicogenomics.” Toxicology in the twentieth century involved animal testing and LD_50_ (Lethal Dose 50) levels where half the population of animals die from the toxin after a certain amount of time, such as in one week or one month. Toxicogenomics in the twenty-first century, in contrast, utilizes modern genetics, epigenetics, and molecular biology technologies.

Toxicogenomics in the twenty-first century should involve more research that utilizes whole-genome sequencing of model-organism or human-stem-cell-derived “organoids,” single-cell analyses, proteomics, complex genetics with conditional-lethal, knock-in or knock-out transgenes, and bioinformatics technologies.

Ideas for research topics in Toxicogenomics include:

- Organoid Toxicology Using Primary Cells and hESC-Derived Tissues.
- Single-Cell Transcriptomic Analyses of Toxicant-Exposed Tissues.
- Single-Cell Epigenomic Analyses of Toxicant-Exposed Tissues.
- Proteomics Technologies of Toxicant-Exposed Tissues.
- Multigenerational and Transgenerational Inheritance of Adaptive Epigenetic Changes of Toxicant-Exposed Model Animals and Humans.
- The Role of Extracellular Vesicles in Transmitting Signals Throughout the Organism After Toxicant Exposure.
- Imaging of Intracellular Alternations Caused By Toxicant Exposure.
- Toxicant-Specific eQTLs (Expression Quantitative Trait Loci).
- Exposures Measurements from Biobanked Tissues, Such as, Neonatal Dried Blood Spots and Shed Teeth.
- Toxicant Effects on the Microbiome.
- Sex-Specific Effects of Toxicants.
- Novel Methods—Second- and third-generation DNA sequencing technologies in Toxicogenomics, GWAS, etc.

In the following sections, we expand upon each of these topics. Hopefully, these topics will inspire future research and publications in these emerging fields in toxicogenomics.

## Organoid toxicology using primary cells and human embryonic stem cell (hESC)-derived tissues

Organoid and organ-on-a-chip technologies are suitable replacement for animal models and will potentially impart multicellular *in vitro* organoid-related information on mechanisms of human toxicity and disease etiology. Organoids have been made from primary cell cultures, differentiated embryonic stem cells, or differentiated induced pluripotent stem cells (iPSC) for the liver (Cao et al., 2017), the heart (Beauchamp et al., 2015; Conant et al., 2017; Devarasetty et al., 2017), the brain (Hunsberger et al., 2015; Schwartz et al., 2015), the kidney (Astashkina et al., 2012; Chuah and Zink, 2017), the testes (Baert et al., 2017), the bladder (Janssen et al., 2013), and potentially multiple other organs and tissues (Xinaris et al., 2015; Ingersoll et al., 2016; Skardal et al., 2016). Liver toxicity, indicated by liver cell death (Weber et al., 2016; Wong et al., 2016), and cardiac toxicity, indicated by changes in heart beat kinetics (Meattini et al., 2017; Yu et al., 2017), account for most drug candidate failures in human trials.

## Single-cell transcriptomic analyses of toxicant-exposed tissues

Transcriptional profiling of various cell types in tissues and their responses to toxicants will impart cell population-specific molecular characterization, specifically identifying target cells and their sensitivity. Typical RNA-seq experiments from tissues are done from populations of hundreds of thousands or millions of cells, but the heterogeneity of the cells are missed. Single-cell RNA sequencing (scRNA-seq) was developed to study tumor heterogeneity (Müller and Diaz, 2017), but it has been used recently to study heterogeneity in other areas, such as, in the immune system (Yang et al., 2017), the brain (Müller and Diaz, 2017; Ofengeim et al., 2017), the retina (Macosko et al., 2015; Quadrato et al., 2017), in embryo development (Mohammed et al., 2017), and in many other tissues (reviewed in Picelli, 2017). Originally, scRNA-seq involved fluorescence activated cell sorting (FACS) or manually dissecting single cells and performing the sequencing reactions in microtiter dishes. However, microfluidics systems have been developed by Fluidigm, Inc., that can sequence either 96 single cells or 800 single cells at a time (www.fluidigm.com). So-called drop-seq technologies were developed that can sequence tens-of-thousands of single cells at a time using single cells captured in femtoliter oil droplets (Macosko et al., 2015). Here is a link for a video on the origins of the drop-seq technique (https://www.youtube.com/watch?v=vL7ptq2Dcf0). The company 10X Genomics have developed a Chromium™ system to perform drop-seq on as many as 10,000 to 100,000 cells at a time, to a depth of up to 50,000 reads per cell (www.10xgenomics.com). Combining scRNA-seq and toxicology can potentially determine how the toxicants affect the distribution of cell types in a tissue.

## Single-cell epigenomic analyses of toxicant-exposed tissues

Drop-seq and other microfluidics technologies described in the previous section have revolutionized the field of single-cell transcriptomics, but these technologies can also be used to study epigenomics at the single-cell level. In 2013, William Greenleaf's laboratory developed assay for transposase-accessible chromatin using sequencing (ATAC-seq) (Buenrostro et al., 2013). This technique can map accessible chromatin by utilizing bacterial Tn5 transposons pre-loaded with primers that can be used for next-generation DNA sequencing. The transposons insert the primers into regions of open chromatin, and bioinformatics approaches, such as, CENTIPEDE, can be used to identify transcription factor motifs in the open chromatin regions (Pique-Regi et al., 2011). Recently, ATAC-seq has been used for mapping the accessible genome of individual cells (scATAC-seq) using the Fluidigm C1™ programmable microfluidics platform (Buenrostro et al., 2015). Combining scATAC-seq with toxicology can be used to identify transcription factors that change their binding characteristics in the presence of toxicants.

## Proteomics technologies of toxicant-exposed tissues

Large scale analysis of gene expression at the protein level will allow unprecedented opportunity to understand toxic mechanisms and/or modes of action and signaling pathways, and identify novel biomarkers of exposure. Post-translational changes, such as phosphorylation, are often induced by toxicants (Caruso et al., 2014). As proteomics technology improves dramatically, smaller and smaller samples can be analyzed for larger and larger numbers of proteins. A top-down approach in proteomics characterizes the entire proteome for changes induced by a toxicant. For example, the changes in the phospho-proteome induced by mercury exposure has been studied in immune cells (Caruso et al., 2014). A PubMed search using the key terms “proteomics” and “toxicology” identifies over 300 published papers going back to 1998. A recent review of the field discusses the role of proteomics to identify adverse outcome pathways for chemical risk assessment (Brockmeier et al., 2017).

## Multigenerational and transgenerational inheritance of adaptive epigenetic changes of toxicant-exposed model animals and humans

Studies on epigenetic transgenerational inheritance will impart new information on how, and the mechanisms by which toxicants may affect multi- and trans-generational inheritance of abnormal developmental phenotypes include epigenetic mis-regulation in germ cells. In 2005, Michael Skinner's laboratory published a seminal paper in this field by showing that rats exposed to the pesticide vinclozolin have behavioral alterations for multiple generations (Anway et al., 2005; Guerrero-Bosagna et al., 2010; Crews et al., 2014). In 2013, Bruce Blumberg's laboratory showed that the anti-fowling agent tributyltin (TBT) is a multigeneration obesogen (Chamorro-García et al., 2013; Janesick et al., 2014). Recently, the Blumberg lab extended these studies showing a male obesity phenotype in the F4 generation, making the results truly transgenerational since the germ cells did not have direct exposure to TBT (Chamorro-Garcia et al., 2017). In 2015, we (Ruden) showed evidence for multigenerational epigenetic inheritance in humans by showing that DNA methylation changes associated with maternal exposure to lead can be transmitted to the grandchildren (Sen et al., 2015). Other laboratories are investigating the role of small RNAs in sperm in the multigeneration inheritance of the obesity phenotype (Cropley et al., 2016). Carvan et al. have demonstrated that mercury-induced epigenetic transgenerational inheritance of abnormal neurobehavior is correlated with sperm epimutations in zebrafish (Carvan et al., 2017). These are all important studies because they can determine the impact of toxicant exposure on future generations in a non-mutagenic manner.

## The role of extracellular vesicles in transmitting signals throughout the organism after toxicant exposure

Extracellular vesicles (EVs) carry small RNA and protein cargos and have recently been shown to signal to different parts of the body after injury. For example, astrocyte-shed extracellular vesicles have been shown to regulate the peripheral leukocyte response to inflammatory brain lesions in an endocrine-like manner (Dickens et al., 2017). Several studies have characterized exosome cargoes that correlate with different brain diseases (Dorsett et al., 2017; Levy, 2017; Selmaj et al., 2017). There have been very few studies linking toxicant exposures to changes in exosomes, but a recent study did a comparative analysis of microRNA and mRNA expression profiles in cells and exosomes under toluene exposure (Lim et al., 2017). We believe that the role that toxicants have on exosome cargoes for endocrine signaling throughout the body is an important emergent field in toxicology research.

## Imaging of intracellular alternations caused by toxicant exposure

Toxicants can cause changes intracellularly, such as, by altering the cytoskeleton or damage the mitochondria, lysosomes, or other organelles. Confocal microscopy, and other high-resolution 3D-imaging techniques, can be used to study intracellular alterations caused by toxicant exposure (Fretaud et al., 2017). Intracellular changes can be studied by fluorescent fusion proteins, such as, green fluorescent protein (GFP) fusions (Walmsley, 2008), or fluorescent molecules, such as, quantum dots (Guo and Liu, 2017; Zhu et al., 2017), or fluorescent DNA dyes (Edward, 2012). Advanced microscopic approaches will always be important in developing toxicogenomic research technologies.

## Toxicant-specific eQTLs (expression quantitative trait loci)

Quantitative traits are phenotypes, such as, height or weight, that vary in a population, usually in a normal distribution. Studies on expression quantitative trait loci (eQTLs) will contribute to variation in gene expression levels and changes in SNPs in response to specific toxicants. In 2009, we (Ruden) published the first paper identifying eQTLs that are induced by developmental lead exposure in Drosophila (Ruden et al., 2009). More recent studies by the Mackay laboratory has identified QTLs involved in lead and cadmium toxicity in the Drosophila model (Zhou et al., 2017). The advantages of using Drosophila in toxicogenomics studies are that it has a short generation time (about 10 days) and that over 205 strains have been sequenced in the Drosophila Genetic Reference Panel (DGRP) (Mackay et al., 2012). There are over five million single-nucleotide polymorphisms (SNPs) in the DGRP collection, and the genome is about 10-fold smaller than the human genome, thereby making the SNP-density in Drosophila, about 1 SNP per ~50 bp, higher than in the entire human sequenced population (5 to 10 SNPs per kb). The DGRP and the power of Drosophila genetics has been underutilized in the toxicogenomics field, but we believe that this model will increasingly show it importance in identifying conserved pathways for toxicant exposure.

## Exposure measurements from biobanked tissues, such as neonatal dried blood spots and shed teeth

Every state in the USA collects neonatal dried bloodspots (NDBS) from every child born in that state for screening for genetic diseases. Michigan and California have tens of millions of NDBS stored in biobanks and are available to biomedical research. We (Ruden) have used this resource to correlate grandmaternal exposure to lead with the grandchildren's DNA methylation pattern (Sen et al., 2015). Teeth are an important emerging resource in toxicology research. Manish Arora, who is both a dentist and a toxicologist, have shown that teeth can be used to study the effects of heavy metal exposure during childhood and the later development of schizophrenia (Modabbernia et al., 2016), and autism spectrum disorder (ASD) (Arora et al., 2017). In the ASD study, monozygotic and dizygotic twins discordant for ASD were used to determine whether fetal and postnatal heavy metal exposure increases ASD risk. They found that ASD cases have reduced uptake of essential elements manganese and zinc, and higher uptake of the neurotoxant lead (Arora et al., 2017). It is important to note that teeth can be used to determine gestational exposures because teeth develop during the second trimester, and there is a distinct physical landmark that develops in the teeth at birth. Arora et al. compare teeth to trees with rings, and laser-scanning mass-spectrometry approaches can be used to determine when and how much lead exposure occurs at the resolution of one or a few weeks. More and more biobanks are starting to collect teeth, such as, in the Environmental Influences on Child Health Outcomes (ECHO) biobank (www.nih.gov/echo), to utilize teeth as an important resource in toxicology studies.

## Toxicant effects on the microbiome

The human microbiome consists of the bacteria that reside in the different parts of the body—both internally and externally (Silbergeld, 2017). The microbiome, which only recently because a field of study because of technological advances in next-generation DNA-sequencing technologies, is increasingly recognized as a critical component in human development, health, disease, and toxicology studies. Its relevance to toxicology involves concepts related to absorption kinetics, metabolism by both the host and the bacteria, and gene-environment pathways of response to the bacterial metabolites that change upon toxicant exposures.

## Sex-specific effects of toxicants

It has long been recognized that toxicants can affect males and females differently. For example, in the tributyltin (TBT) example discussed in a previous section, only the F1, F2, and F3 male offspring are made obese by the TBT obesogen (Chamorro-García et al., 2013; Janesick et al., 2014). Chemical compounds such as TBT, which makes a three-ring structure, are endocrine disruptors that affect signaling by steroid hormone receptors and transcription factors such as PPAR gamma. Since the male and female endocrine systems are very different from each other, with males making primarily testosterone and females making primarily estrogen, for instance, it is important to study the effects of sex in toxicological studies.

## Novel methods—second- and third-generation DNA sequencing technologies in toxicogenomics, GWAS, etc

New methods are being developed every year that affect the field of toxicogenomics. DNA methylation can be studied by using a chemical trick—bisulfite converts unmethylated cytosines to uracil, but does not convert methylated cytosine as efficiently. However, other DNA modifications have been identified, such as, N6-mA, which can be studied best by single-molecule DNA-sequencing technologies, such as, the Pacific Biosciences SMART-seq™ technology (Flusberg et al., 2010). How toxicants might affect N6-mA or other base modifications is an unexplored area of toxicogenomics research. Other technologies are just beginning to be used in toxicology research, such as, genome-wide association (GWAS) analyses. The Mackay laboratory have used the Drosophila Genome Reference Panel (DGRP) to conduct a GWAS analysis on the toxicology of lead and cadmium (Zhou et al., 2016). GWAS studies in humans have also been used to characterize human genetic susceptibility for environmental chemical risk assessment (Yang et al., 2012; Mortensen and Euling, 2013). More studies such as, these are needed to expand toxicogenomics research in the twenty-first century.

## Conclusions and future studies in toxicogenomics

**Figure 1:**
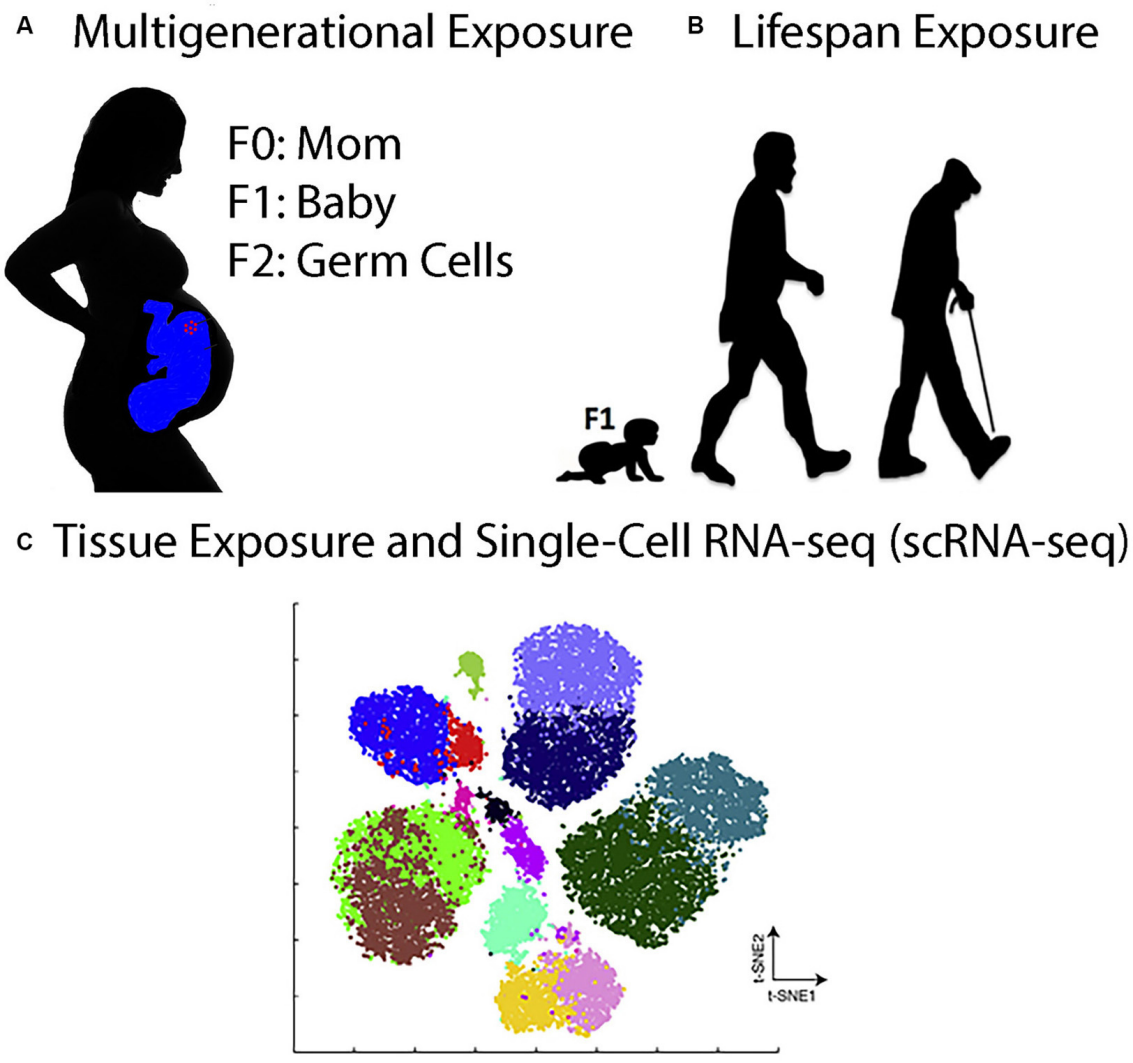
Toxicogenomics Experiments in the Twenty-First Century. **(A)**, Multigenerational exposure toxicogenomics involves studying the effects of toxicant exposures in pregnant women (F0) on her babies (F1) and on her grandchildren (F2) who were exposed as germline stem cells in the pregnant mothers. **(B)** Lifespan exposure toxicogenomics involves studying the effects of toxicant exposures anytime during the life of an organism, from babies to adults to the elderly. **(C)** Tissue exposure and single-cell RNA-seq (scRNA-seq) involve exposing organisms or 3D organoids to toxicants and then analyzing the gene expression pattern in all of the cells individually. The figure shows a t-Distributed Stochastic Neighbor Embedding (t-SNE) analysis of hypothetical scRNA-seq data. The t-SNE analysis is a technique for dimensionality reduction that is well suited for the visualization of high-dimensional datasets (Taskesen and Reinders, 2016). Each cluster corresponds to one cell type in the tissue being analyzed.

Perhaps the greatest challenge facing the fields of toxicology and toxicogenomics in the future is to produce, process, curate, archive, and analyze immense genomic, proteomics, and image datasets at the multi-generational, lifespan, and single-cell levels (Figure 1). To take just one example, a single scRNA-seq experiment that analyzes the transcriptome of 100,000 cells ± toxicant exposure can generate in 1 week over 10 terabytes (TB) of raw sequencing data on the NovaSeq™ next-generation DNA sequencing platform from Illumina and can take over a week to align the data to a reference genome on a typical 1,000-node supercomputer cluster (www.illumina.com). In one year, this would generate ~0.5 petabytes (PB) of raw sequencing data. Clearly, this exceeds the resources and computer expertise at most universities, for just this one type of experiment.

In the new and emerging field of toxicogenomics, no single person has the expertise or the time to effectively bring these components together. As technologies continue to improve, success in advancing toxicogenomics will depend on collaborations across large trans-disciplinary and multi-disciplinary groups. Experts who generate genomic and epigenomic data are needed to produce the raw genomic data for subsequent analysis. As the typical equipment for next-generation sequencing is in the $1 million range, it is beyond what an individual investigator can afford. Therefore, core facilities, such as, genomics and proteomics cores, are needed to be expanded and modernized at universities to meet the needs of the entire research community. In this model, research done by the individual investigators in their own laboratories will be reduced, as research in core facilities expands.

Bioinformaticians and computer scientists are needed to provide programming and computer science expertise to efficiently process, curate, archive, and analyze vast genomic datasets, and to effectively utilize existing high performance computing resources. Newer and better high performance computing resources need to be improved on an annual basis to meet the expanding demand.

Modelers, mathematicians, and computer scientists will be needed to work with the bioinformaticians to develop new ways to not only process data to a usable format, but also to image and visualize data. For example, we (Ruden) have worked with computer scientists to develop the SnpEff (Cingolani et al., 2012b) software, which is used to characterize the effects of SNPs in whole-genome sequencing data, and has been cited over 1,700 times since it came out in 2012. As of May/June 2017, this highly cited paper received enough citations to place it in the top 1% of the academic field of Molecular Biology & Genetics based on a highly cited threshold for the field and publication year. Also in our laboratory, the SnpSift (Cingolani et al., 2012a) software was developed about the same as SnpEff to identify individual causative SNPs in Drosophila genetic screens. As a third example, CloudAligner (Nguyen et al., 2011), a MapReduce based tool to align read onto reference genome using cloud computing resources, such as the Amazon Cloud™, has been developed in a collaboration between Weisong Shi in the Computer Science Department and our laboratory (Ruden).

It might be more cost effective for most universities to use computing resources on the cloud rather than purchasing their own computer systems. Similarly, it might be more cost effective for investigators to explore companies outside of the university to conduct next-generation sequencing experiments, such as, with the company GeneWiz (www.genewiz.com), which can provide services from library preparation and sequencing to detailed bioinformatics analyses. Clearly, new ways of thinking about how to conduct toxicogenomics experiments is needed as the field develops and new technologies emerge.

## Author contributions

DR: Wrote the first draft; KG: Edited and added a section; MA: Edited and commented on all drafts.

### Conflict of interest statement

The authors declare that the research was conducted in the absence of any commercial or financial relationships that could be construed as a potential conflict of interest.

## References

[B1] Anway M. D. Cupp A. S. Uzumcu M. Skinner M. K. (2005). Epigenetic transgenerational actions of endocrine disruptors and male fertility. Science 308, 1466–1469. 10.1126/science.1108190 15933200 PMC11423801

[B2] Arora M. Reichenberg A. Willfors C. Austin C. Gennings C. Berggren S.. (2017). Fetal and postnatal metal dysregulation in autism. Nat. Commun. 8:15493. 10.1038/ncomms15493 28569757 PMC5461492

[B3] Astashkina A. I. Mann B. K. Prestwich G. D. Grainger D. W. (2012). A 3-D organoid kidney culture model engineered for high-throughput nephrotoxicity assays. Biomaterials 33, 4700–4711. 10.1016/j.biomaterials.2012.02.063 22444643

[B4] Baert Y. De Kock J. Alves-Lopes J. P. Söder O. Stukenborg J. B. Goossens E. (2017). Primary human testicular cells self-organize into organoids with testicular properties. Stem Cell Rep. 8, 30–38. 10.1016/j.stemcr.2016.11.012 28017656 PMC5233407

[B5] Beauchamp P. Moritz W. Kelm J. M. Ullrich N. D. Agarkova I. Anson B. D.. (2015). Development and characterization of a scaffold-free 3D spheroid model of induced pluripotent stem cell-derived human cardiomyocytes. Tissue Eng. C Methods 21, 852–861. 10.1089/ten.tec.2014.0376 25654582

[B6] Brockmeier E. K. Hodges G. Hutchinson T. H. Butler E. Hecker M. Tollefsen K. E.. (2017). The role of omics in the application of adverse outcome pathways for chemical risk assessment. Toxicol Sci. 158, 252–262. 10.1093/toxsci/kfx097 28525648 PMC5837273

[B7] Buenrostro J. D. Giresi P. G. Zaba L. C. Chang H. Y. Greenleaf W. J. (2013). Transposition of native chromatin for fast and sensitive epigenomic profiling of open chromatin, DNA-binding proteins and nucleosome position. Nat. Methods 10, 1213–1218. 10.1038/nmeth.2688 24097267 PMC3959825

[B8] Buenrostro J. D. Wu B. Litzenburger U. M. Ruff D. Gonzales M. L. Snyder M. P.. (2015). Single-cell chromatin accessibility reveals principles of regulatory variation. Nature 523, 486–490. 10.1038/nature14590 26083756 PMC4685948

[B9] Cao W. Chen K. Bolkestein M. Yin Y. M. Verstegen M. A. M. M. Bijvelds J. C.. (2017). Dynamics of proliferative and quiescent stem cells in liver homeostasis and injury. Gastroenterology 153, 1133–1147. 10.1053/j.gastro.2017.07.006 28716722

[B10] Caruso J. A. Stemmer P. M. Dombkowski A. Caruthers N. J. Gill R. Rosenspire A. J. (2014). A systems toxicology approach identifies Lyn as a key signaling phosphoprotein modulated by mercury in a B lymphocyte cell model. Toxicol. Appl. Pharmacol. 276, 47–54. 10.1016/j.taap.2014.01.002 24440445 PMC4005802

[B11] Carvan M. J. Kalluvila T. A. Klingler R. H. Larson J. K. Pickens M. Mora-Zamorano F. X.. (2017). Mercury-induced epigenetic transgenerational inheritance of abnormal neurobehavior is correlated with sperm epimutations in zebrafish. PLoS ONE 12:e0176155. 10.1371/journal.pone.0176155 28464002 PMC5413066

[B12] Chamorro-Garcia R. Diaz-Castillo C. Shoucri B. M. Kaech H. Leavitt R. Shioda T. (2017). Ancestral perinatal obesogen exposure results in a transgenerational thrifty 2 phenotype in mice. bioRvix. 1–35. 10.1101/201384 PMC5722856 29222412

[B13] Chamorro-García R. Sahu M. Abbey R. J. Laude J. Pham N. Blumberg B. (2013). Transgenerational inheritance of increased fat depot size, stem cell reprogramming, and hepatic steatosis elicited by prenatal exposure to the obesogen tributyltin in mice. Environ. Health Perspect. 121, 359–366. 10.1289/ehp.1205701 23322813 PMC3621201

[B14] Chuah J. K. Zink D. (2017). Stem cell-derived kidney cells and organoids: recent breakthroughs and emerging applications. Biotechnol. Adv. 35, 150–167. 10.1016/j.biotechadv.2016.12.001 28017905

[B15] Cingolani P. Patel V. M. Coon M. Nguyen T. Land S. J. Ruden D. M.. (2012a). Using *Drosophila melanogaster* as a model for genotoxic chemical mutational studies with a new program, SnpSift. Front. Genet. 3:35. 10.3389/fgene.2012.00035 22435069 PMC3304048

[B16] Cingolani P. Platts A. L. Wang le L. Coon M. Nguyen T. Wang L. Land S. J.. (2012b). A program for annotating and predicting the effects of single nucleotide polymorphisms, SnpEff: SNPs in the genome of *Drosophila melanogaster* strain w1118; iso-2; iso-3. Fly 6, 80–92. 10.4161/fly.19695 22728672 PMC3679285

[B17] Conant G. Lai B. F. L. Lu R. X. Z. Korolj A. Wang E. Y. Radisic M. (2017). High-content assessment of cardiac function using heart-on-a-chip devices as drug screening model. Stem Cell Rev. 13, 335–346. 10.1007/s12015-017-9736-2 28429185

[B18] Crews D. Gillette R. Miller-Crews I. Gore A. C. Skinner M. K. (2014). Nature, nurture and epigenetics. Mol. Cell. Endocrinol. 398, 42–52. 10.1016/j.mce.2014.07.013 25102229 PMC4300943

[B19] Cropley J. E. Eaton S. A. Aiken A. Young P. E. Giannoulatou E. Ho J. W.. (2016). Male-lineage transmission of an acquired metabolic phenotype induced by grand-paternal obesity. Mol. Metab. 5, 699–708. 10.1016/j.molmet.2016.06.008 27656407 PMC5021672

[B20] Devarasetty M. Forsythe S. Shupe T. D. Soker S. Bishop C. E. Atala A.. (2017). Optical tracking and digital quantification of beating behavior in bioengineered human cardiac organoids. Biosensors 7:E24. 10.3390/bios7030024 28644395 PMC5618030

[B21] Dickens A. M. Tovar-Y-Romo L. B. Yoo S. W. Trout A. L. Bae M. Kanmogne M.. (2017). Astrocyte-shed extracellular vesicles regulate the peripheral leukocyte response to inflammatory brain lesions. Sci. Signal. 10:eaai7696. 10.1126/scisignal.aai7696 28377412 PMC5590230

[B22] Dorsett C. R. McGuire J. L. Niedzielko T. L. DePasquale E. A. Meller J. Floyd C. L.. (2017). Traumatic brain injury induces alterations in cortical glutamate uptake without a reduction in glutamate transporter-1 protein expression. J. Neurotrauma 34, 220–234. 10.1089/neu.2015.4372 27312729 PMC5198172

[B23] Edward R. (2012). Red/far-red fluorescing DNA-specific anthraquinones for nucl:cyto segmentation and viability reporting in cell-based assays. Methods Enzymol. 505, 23–45. 10.1016/B978-0-12-388448-0.00010-3 22289446

[B24] Flusberg B. A. Webster D. R. Lee J. H. Travers K. J. Olivares E. C. Clark T. A.. (2010). Direct detection of DNA methylation during single-molecule, real-time sequencing. Nat. Methods 7, 461–465. 10.1038/nmeth.1459 20453866 PMC2879396

[B25] Frétaud M. Rivière L. Job É. Gay S. Lareyre J. J. Joly J. S.. (2017). High-resolution 3D imaging of whole organ after clearing: taking a new look at the zebrafish testis. Sci. Rep. 7:43012. 10.1038/srep43012 28211501 PMC5314416

[B26] Guerrero-Bosagna C. Settles M. Lucker B. Skinner M. K. (2010). Epigenetic transgenerational actions of vinclozolin on promoter regions of the sperm epigenome. PLoS ONE 5:e13100. 10.1371/journal.pone.0013100 20927350 PMC2948035

[B27] Guo D. Liu R. (2017). Spectroscopic investigation of the effects of aqueous-phase prepared CdTe quantum dots on protein hemoglobin at the molecular level. J. Biochem. Mol. Toxicol. 31:e21953. 10.1002/jbt.21953 28661553

[B28] Hunsberger J. G. Efthymiou A. G. Malik N. Behl M. Mead I. L. Zeng X.. (2015). Induced pluripotent stem cell models to enable *in vitro* models for screening in the central nervous system. Stem Cells Dev. 24, 1852–1864. 10.1089/scd.2014.0531 25794298 PMC4533087

[B29] Ingersoll T. Cole S. Madren-Whalley J. Booker L. Dorsey R. Li A.. (2016). Generalized additive mixed-models for pharmacology using integrated discrete multiple organ co-culture. PLoS ONE 11:e0152985. 10.1371/journal.pone.0152985 27110941 PMC4844122

[B30] Janesick A. S. Shioda T. Blumberg B. (2014). Transgenerational inheritance of prenatal obesogen exposure. Mol. Cell. Endocrinol. 398, 31–35. 10.1016/j.mce.2014.09.002 25218215 PMC4262625

[B31] Janssen D. A. Geutjes P. J. Odenthal J. van Kuppevelt T. H. Schalken J. A. Feitz W. F.. (2013). A new, straightforward *ex vivo* organoid bladder mucosal model for preclinical research. J. Urol. 190, 341–349. 10.1016/j.juro.2012.12.103 23306090

[B32] Levy E. (2017). Exosomes in the diseased brain: first insights from *in vivo* studies. Front. Neurosci. 11:142. 10.3389/fnins.2017.00142 28386213 PMC5362612

[B33] Lim J. H. Song M. K. Cho Y. Kim W. Han S. O. Ryu J. C. (2017). Comparative analysis of microRNA and mRNA expression profiles in cells and exosomes under toluene exposure. Toxicol. In Vitro 41, 92–101. 10.1016/j.tiv.2017.02.020 28245982

[B34] Mackay T. F. Richards S. Stone E. A. Barbadilla A. Ayroles J. F. Zhu D.. (2012). The drosophila melanogaster genetic reference panel. Nature 482, 173–178. 10.1038/nature10811 22318601 PMC3683990

[B35] Macosko E. Z. Basu A. Satija R. Nemesh J. Shekhar K. Goldman M.. (2015). Highly parallel genome-wide expression profiling of individual cells using nanoliter droplets. Cell 161, 1202–1214. 10.1016/j.cell.2015.05.002 26000488 PMC4481139

[B36] Meattini I. Curigliano G. Terziani F. Becherini C. Airoldi M. Allegrini G. (2017). SAFE trial: an ongoing randomized clinical study to assess the role of cardiotoxicity prevention in breast cancer patients treated with anthracyclines with or without trastuzumab. Med. Oncol. 34:75 10.1007/s12032-017-0938-x 28364270

[B37] Modabbernia A. Velthorst E. Gennings C. De Haan L. Austin C. Sutterland A.. (2016). Early-life metal exposure and schizophrenia: a proof-of-concept study using novel tooth-matrix biomarkers. Eur. Psychiatry 36, 1–6. 10.1016/j.eurpsy.2016.03.006 27311101 PMC5300790

[B38] Mohammed H. Hernando-Herraez I. Savino A. Scialdone A. Macaulay I. Mulas C.. (2017). Single-cell landscape of transcriptional heterogeneity and cell fate decisions during mouse early gastrulation. Cell Rep. 20, 1215–1228. 10.1016/j.celrep.2017.07.009 28768204 PMC5554778

[B39] Mortensen H. M. Euling S. Y. (2013). Integrating mechanistic and polymorphism data to characterize human genetic susceptibility for environmental chemical risk assessment in the 21st century. Toxicol. Appl. Pharmacol. 271, 395–404. 10.1016/j.taap.2011.01.015 21291902

[B40] Müller S. Diaz A. (2017). Single-Cell mRNA sequencing in cancer research: integrating the genomic fingerprint. Front. Genet. 8:73. 10.3389/fgene.2017.00073 28620412 PMC5450061

[B41] Nguyen T. Shi W. Ruden D. (2011). CloudAligner: a fast and full-featured mapreduce based tool for sequence mapping. BMC Res. Notes 4:171. 10.1186/1756-0500-4-171 21645377 PMC3127959

[B42] Ofengeim D. Giagtzoglou N. Huh D. Zou C. Yuan J. (2017). Single-Cell RNA sequencing: unraveling the brain one cell at a time. Trends Mol. Med. 23, 563–576. 10.1016/j.molmed.2017.04.006 28501348 PMC5531055

[B43] Picelli S. (2017). Single-cell RNA-sequencing: the future of genome biology is now. RNA Biol. 14, 637–650. 10.1080/15476286.2016.1201618 27442339 PMC5449089

[B44] Pique-Regi R. Degner J. F. Pai A. A. Gaffney D. J. Gilad Y. Pritchard J. K. (2011). Accurate inference of transcription factor binding from DNA sequence and chromatin accessibility data. Genome Res. 21, 447–455. 10.1101/gr.112623.110 21106904 PMC3044858

[B45] Quadrato G. Nguyen T. Macosko E. Z. Sherwood J. L. Min Yang S. Berger D. R.. (2017). Cell diversity and network dynamics in photosensitive human brain organoids. Nature 545, 48–53. 10.1038/nature22047 28445462 PMC5659341

[B46] Ruden D. M. Chen L. Possidente D. Possidente B. Rasouli P. Wang L.. (2009). Genetical toxicogenomics in Drosophila identifies master-modulatory loci that are regulated by developmental exposure to lead. Neurotoxicology 30, 898–914. 10.1016/j.neuro.2009.08.011 19737576 PMC2789871

[B47] Schwartz M. P. Hou Z. Propson N. E. Zhang J. Engstrom C. J. Santos Costa V.. (2015). Human pluripotent stem cell-derived neural constructs for predicting neural toxicity. Proc. Natl. Acad. Sci. U.S.A. 112, 12516–12521. 10.1073/pnas.1516645112 26392547 PMC4603492

[B48] Selmaj I. Mycko M. P. Raine C. S. Selmaj K. W. (2017). The role of exosomes in CNS inflammation and their involvement in multiple sclerosis. J. Neuroimmunol. 306, 1–10. 10.1016/j.jneuroim.2017.02.002 28385180

[B49] Sen A. Heredia N. Senut M. C. Land S. Hollocher K. Lu X.. (2015). Multigenerational epigenetic inheritance in humans: DNA methylation changes associated with maternal exposure to lead can be transmitted to the grandchildren. Sci. Rep. 5:14466. 10.1038/srep14466 26417717 PMC4586440

[B50] Silbergeld E. K. (2017). The microbiome. Toxicol. Pathol. 45, 190–194. 10.1177/0192623316672073 27770110

[B51] Skardal A. Shupe T. Atala A. (2016). Organoid-on-a-chip and body-on-a-chip systems for drug screening and disease modeling. Drug Discov. Today 21, 1399–1411. 10.1016/j.drudis.2016.07.003 27422270 PMC9039871

[B52] Taskesen E. Reinders M. J. (2016). 2D Representation of transcriptomes by t-SNE Exposes relatedness between human tissues. PLoS ONE 11:e0149853. 10.1371/journal.pone.0149853 26906061 PMC4764374

[B53] Walmsley R. M. (2008). GADD45a-GFP GreenScreen HC genotoxicity screening assay. Expert Opin. Drug Metab. Toxicol. 4, 827–835. 10.1517/17425255.4.6.827 18611122

[B54] Weber J. S. Postow M. Lao C. D. Schadendorf D. (2016). Management of adverse events following treatment with anti-programmed death-1 agents. Oncologist 21, 1230–1240. 10.1634/theoncologist.2016-0055 27401894 PMC5061539

[B55] Wong H. H. Barton C. Acton G. McLeod R. Halford S. (2016). Trends in the characteristics, dose-limiting toxicities and efficacy of phase I oncology trials: the cancer research UK experience. Eur. J. Cancer 66, 9–16. 10.1016/j.ejca.2016.07.004 27514008

[B56] Xinaris C. Brizi V. Remuzzi G. (2015). Organoid models and applications in biomedical research. Nephron 130, 191–199. 10.1159/000433566 26112599

[B57] Yang J. Tanaka Y. Seay M. Li Z. Jin J. Garmire L. X.. (2017). Single cell transcriptomics reveals unanticipated features of early hematopoietic precursors. Nucleic Acids Res. 45, 1281–1296. 10.1093/nar/gkw1214 28003475 PMC5388401

[B58] Yang X. Zhang B. Zhu J. (2012). Functional genomics- and network-driven systems biology approaches for pharmacogenomics and toxicogenomics. Curr. Drug Metab. 13, 952–967. 10.2174/138920012802138633 22591344

[B59] Yu A. F. Mukku R. B. Verma S. Liu J. E. Oeffinger K. C. Steingart R. M.. (2017). Cardiac safety of non-anthracycline trastuzumab-based therapy for HER2-positive breast cancer. Breast Cancer Res. Treat. 166, 241–247. 10.1007/s10549-017-4362-x 28710537 PMC5647229

[B60] Zhou S. Luoma S. E. St Armour G. E. Thakkar E. Mackay T. F. C. Anholt R. H. (2017). A drosophila model for toxicogenomics: genetic variation in susceptibility to heavy metal exposure. PLoS Genet. 13:e1006907. 10.1371/journal.pgen.1006907 28732062 PMC5544243

[B61] Zhou S. Morozova T. V. Hussain Y. N. Luoma S. E. McCoy L. Yamamoto A.. (2016). The genetic basis for variation in sensitivity to lead toxicity in drosophila melanogaster. Environ. Health Perspect. 124, 1062–1070. 10.1289/ehp.1510513 26859824 PMC4937873

[B62] Zhu X. Wu G. Lu N. Yuan X. Li B. (2017). A miniaturized electrochemical toxicity biosensor based on graphene oxide quantum dots/carboxylated carbon nanotubes for assessment of priority pollutants. J. Hazard. Mater. 324, 272–280. 10.1016/j.jhazmat.2016.10.057 27810324

